# Participatory Systems Thinking to Elucidate Drivers of Food Access and Diet Disparities among Minoritized Urban Populations

**DOI:** 10.1007/s11524-024-00895-3

**Published:** 2024-07-24

**Authors:** Brent A. Langellier, Sofia Argibay, Rosie Mae Henson, Caroline Kravitz, Alexandra Eastus, Ivana Stankov, Irene Headen

**Affiliations:** 1 Department of Health Management and Policy, Dornsife School of Public Health, Drexel University, 3215 Market St, 3rd Floor, Office 356, Philadelphia, PA 19104 USA; 2 UniSA Allied Health and Human Performance, University of South Australia, Adelaide, Australia; 3 Urban Health Collaborative, Dornsife School of Public Health, Drexel University, Philadelphia, PA USA; 4 Department of Community Health and Prevention, Dornsife School of Public Health, Drexel University, Philadelphia, PA USA

**Keywords:** Health disparities, Diet, Urban health, Racism, Food environment

## Abstract

The purpose of this study was to use participatory systems thinking to develop a dynamic conceptual framework of racial/ethnic and other intersecting disparities (e.g., income) in food access and diet in Philadelphia and to identify policy levers to address these disparities. We conducted three group model building workshops, each consisting of a series of scripted activities. Key artifacts or outputs included qualitative system maps, or causal loop diagrams, identifying the variables, relationships, and feedback loops that drive diet disparities in Philadelphia, Pennsylvania. We used semi-structured methods informed by inductive thematic analysis and network measures to synthesize findings into a single causal loop diagram. There were twenty-nine participants with differing vantages and expertise in Philadelphia’s food system, broadly representing the policy, community, and research domains. In the synthesis model, participants identified 14 reinforcing feedback loops and one balancing feedback loop that drive diet and food access disparities in Philadelphia. The most highly connected variables were upstream factors, including those related to racism (e.g., residential segregation) and community power (e.g., community land control). Consistent with existing frameworks, addressing disparities will require a focus on upstream social determinants. However, existing frameworks should be adapted to emphasize and disrupt the interdependent, reinforcing feedback loops that maintain and exacerbate disparities in fundamental social causes. Our findings suggest that promising policies include those that empower minoritized communities, address socioeconomic inequities, improve community land control, and increase access to affordable, healthy, and culturally meaningful foods.

**Supplementary Information:**

The online version contains supplementary material available at 10.1007/s11524-024-00895-3.

## Introduction

Racial/ethnic disparities in food insecurity in the USA are large and have persisted over time [1]. In 2021, 19.8% of Black and 16.2% of Hispanic households were food insecure, compared to just 7% of White households. Food insecurity prevalence increased in 2022, largely as a consequence of economic disruption as part of the COVID-19 pandemic, but racial/ethnic disparities persisted. Food insecurity—or inadequate access to sufficient healthy, nutritious, and culturally appropriate food—is associated with unhealthy diets and increased risk of several health conditions, including poor self-rated physical and mental health and increased incidence of chronic disease risk factors including hypertension and diabetes [2].

Racial/ethnic disparities in food security, food access, and diet are likely driven by the interplay of social inequities overlayed on an inequitable food system. Racial/ethnic disparities in income are likely a key contributor. For example, 27% of households with incomes below 185% of the federal poverty level (FPL; $55,500 for a family of four in 2024) are food insecure compared to just 5% with incomes above 185% of the FPL [3, 4]. Income inequality likely also contributes to inequities in diet healthfulness due to the low price of energy-dense, non-nutritious food relative to healthier alternatives [5, 6].

Urban environments provide insights into the entangled nature of food and social systems. For example, disparities in healthy food access likely result from high levels of racial/ethnic residential segregation combined with the inequitable distribution of food retailers across predominantly White and minoritized neighborhoods [7, 8]. Research conducted in Philadelphia has shown that grocery stores located in neighborhoods with higher proportions of Black residents have less food choice diversity and fewer healthy food options than those in White neighborhoods [9]. As a consequence, residents in predominantly Black neighborhoods must travel further to access sufficient food options or, alternatively, must settle for less healthful foods available closer to home compared to residents in predominantly White neighborhoods [9].

The consistency of food insecurity disparities across heterogeneous spatial contexts (e.g., across different cities) and their persistence through time suggest that disparities are an emergent consequence of a complex and dynamic system [10]. Several existing frameworks appropriately emphasize that disparities are driven by factors across multiple domains (e.g., biological, behavioral, physical/built environment) and levels (i.e., individual, interpersonal, community, societal) of influence [11]. This multiscale, multilevel structure is consistent with a complex systems perspective. A further hallmark of complex systems, which is not as well integrated into existing frameworks, is the presence of feedback loops or closed chains of causal influence. Reinforcing feedback loops accelerates the direction of changes made to a system and can be either virtuous or vicious cycles depending on whether the direction of change is desirable. There are almost certainly important reinforcing feedback loops (e.g., cycles of poverty) that accelerate existing social disadvantages. In contrast, balancing feedback loops are goal-seeking or stabilizing loops that often act as a brake on changes made to a system. Balancing loops can be good or bad—depending on what goal they are seeking or what is being stabilized—and in the context of health and social disparities may help explain why previous well-intentioned, well-thought-out interventions have had limited success [12–14].

Quantitative and qualitative complex systems methods can help advance understanding of the structure and function of systems that cause disparities in food insecurity, diet, and health [10, 15]. A relevant example is social scientists’ use of agent-based simulation models, a quantitative systems approach, to examine the social dynamics that lead to residential segregation and the health impacts of segregated cities [16–19]. System dynamics modeling is an approach that can be either quantitative or qualitative and involves the development of causal diagrams and/or computer simulation models that portray processes of accumulation and feedback. The qualitative branch—which includes both community-based system dynamics modeling and group model building—uses stakeholder engagement methods to map and understand the structure and function of a complex system from the perspectives of stakeholders knowledgeable about the system under study [20]. Group model building typically involves a series of scripted activities that introduce key concepts in systems thinking and then provide participants with tools and support to make their mental models (cognitive representations of a system) explicit, to talk through differences in mental models across stakeholders, and to identify leverage points to improve system outputs. Group model building can help participants develop a shared understanding of a complex problem, view a problem from a systems thinking perspective (e.g., by focusing on feedback loops), and build consensus about the changes needed to address issues within the system [20].

Previous studies have used group model building to elucidate the structure and function of systems related to food and diet [21–24], but far fewer have focused on food and diet disparities [22]. The primary example of a study focused on disparities was conducted by Freedman et al., who used group model building as part of a mixed-methods study to examine the complexity and inequity of food systems in historically redlined neighborhoods in Cleveland, Ohio [22]. The study identified 10 feedback mechanisms driving food system inequities, broadly organized into three domains: meeting basic food needs with dignity (i.e., side hustle, government benefits, emergency food assistance, stigma, and stereotypes), local food supply and demand dynamics (i.e., healthy food retail, job security, food culture, and norms), and community empowerment and food sovereignty (i.e., community power, urban agriculture, risk of gentrification). The feedback loops identified by the study identify leverage points for policy interventions to advance nutrition equity and demonstrate the interconnected nature of food and social systems. In the current study, we complement prior work by Freedman and others by focusing on systems that drive racial/ethnic disparities in a different urban environment, Philadelphia, Pennsylvania. In addition to elucidating specific system structures that cause disparities in Philadelphia, this study seeks to generate initial insights into the mechanisms (e.g., causal relationships, feedback loops) responsible for generating and maintaining disparities across varying urban contexts.

We describe the main results of a series of three group model building workshops to elucidate the structure and function of the systems that cause disparities in food access and diet. Though our primary focus was on racial/ethnic disparities, we did not specifically limit prompts to focus exclusively on race/ethnicity to allow for the exploration of disparities based on other social identities that intersect with race/ethnicity (e.g., income, power). The objectives of the study were to convene and engage stakeholders, to provide a common language and understanding to help make their mental models explicit, to work toward a common understanding of the structure and function of the system, and to identify promising policies and interventions that incorporate systems thinking and feedback perspectives.

## Methods

### Participant Recruitment

We identified and recruited participants using purposive and snowball sampling based on a list of policy, community, and research stakeholders and organizations from different vantage points in the food system. This includes people with lived experience of food insecurity and representing different neighborhoods and social groups across the city. Participants were offered compensation of $500 for participating in one 8-h workshop.

### Group Model Building Overview

The study was designed by a core modeling team comprised of three members of the research team with previous experience designing and implementing group model building studies and one research team member who led participant recruitment. Broadly, the study consisted of three 1-day workshops, each with a unique group of participants. Three workshops were conducted to allow for the participation of diverse stakeholders, as well as considerations regarding the study budget and timeline. We audio-recorded all workshops and activities and used transcripts in subsequent analyses (described below). Additionally, multiple note-takers were present in each session and took notes to further describe and clarify the development of workshop artifacts. The Drexel University Institutional Review Board reviewed and approved the study protocol.

Table 1 is a sample agenda for one of the workshops, including key scripted activities and outputs; Appendix 1 is a sample facilitation manual. After conducting an icebreaker exercise to allow participants to air their hopes and fears about the session, we implemented the Graphs Over Time script, in which each participant identified one or more variables that influence diet disparities in Philadelphia and then drew both a “hoped for” and a “feared” trajectory describing how each variable might change over time. Graphs Over Time is generative in that it prompts the group to generate a list of important variables; it also encourages participants to think dynamically (i.e., temporal trajectories) and counterfactually (i.e., hoped-for vs. feared trajectories). In the next key activity, we asked small groups of 3–4 participants to develop a causal loop diagram (CLD) depicting variables, relationships, and feedback loops that explained diet disparities in Philadelphia.

**Table 1:** Sample agenda for a group model building workshop

Activity	Artifacts
Hopes and fears	List of hopes and fears
Graphs over time	Graphs over time; clusters/themes
Causal loop diagramming	2–3 CLDs
Model synthesis	Synthesis CLDs; identification of common structures/variables; example of CLD function
Action ideas	List of action ideas, ranked by feasibility, potential impact

After each group presented their CLD back to the larger group, the facilitation team drafted an aggregate model that incorporated the small group CLDs. The goal in drafting the aggregate model was to include all feedback loops identified in each of the small group CLDs, merge common elements (e.g., variables that were the same or very similar), and make only minimal (and ideally no) changes to structures included in the small group CLDs. The full group then engaged in a synthesis activity; the goal of the synthesis activity was to edit the aggregate model and, ultimately, to achieve consensus that it accurately incorporated stakeholders’ perspectives. While the activity was guided by facilitators, changes to the model were made directly by participants. Though disagreements were uncommon, the facilitators asked clarifying questions to elicit further descriptions and support participants to productively work through any disagreements. In the final key activity, we asked each participant to propose intervention ideas to eliminate diet disparities. Additional details regarding group model building methods, scripted activities, and roles are described in detail elsewhere [25].

### Artifact Analysis

The research team used a semi-structured approach to generate a single, synthesis model that incorporates key variables, relationships, and feedback loops from the aggregate CLDs produced in each of the three workshops. First, we conducted a content analysis to identify common themes among the variables in the aggregate CLDs produced in each of the three workshops. We adopted an analytic approach informed by Pluchinotta and colleagues [26], which draws from well-established qualitative research methods, particularly inductive thematic analysis [27]. We decomposed each of the three aggregate CLDs into a list of unique variables and then used an inductive thematic analysis to sort each variable into a single, “best fit” cluster or theme using workshop notes and transcripts. Three coders—all present in the workshops—independently identified and named the clusters and then discussed and reconciled divergent results (Braun & Clarke, 2006).

Second, we calculated the degree centrality for all variables in each of the three aggregate CLDs in order to identify variables that were highly connected within the context of the system structures identified by participants [28]. For each variable, we counted the number of CLDs in which the variable was present (i.e., range of only one CLD to all three) and the number of causal connections to the variable (i.e., the total number of causal arrows into and out of each variable) across each of the three CLDs [28].

We used the content analysis, workshop transcripts and recordings, and degree centrality of each variable as inputs in prioritizing loops and variables to be included in the synthesis CLD. We used procedures similar to rigorously interpreted quotation analysis (RIQ) [29] to synthesize variables, connections, and feedback loops across workshops. RIQ uses an interpretive process to confirm or disconfirm all diagram elements in a CLD by comparing it to stakeholders’ explicit descriptions of their experiences, based primarily on transcripts, recordings, and notes from the workshops. We decomposed participant quotations into small phrases and identified phrases that supported or disconfirmed CLD elements (variables and feedback loops). For example, a code assigned to a causal arrow can confirm the direction of the relationship between the variables as well as the directionality (i.e., positive or negative). When necessary, we made minor revisions to variable names, variable definitions, causal arrows, and their polarity to accurately reflect participant quotations and discussions. We conducted the synthesis iteratively, with three members of the research team making synthesis decisions and presenting participant quotations as evidence to the full team until we reached a consensus. We repeated this process until the synthesized CLD represented the key interrelationships and main feedback loops identified by stakeholders.

## Results

### Stakeholder Participation

Twenty-nine stakeholders participated across the three workshops (*n* = 7, 9, and 13, respectively). Five participants were researchers, nine were policy experts, and 15 represented community-based organizations or community members active in food justice. Participant breakdown by workshop is in Supplemental Table 1.

### Common Understanding of the System

The aggregate CLDs produced in each of the three workshops contained a total of 56 unique variables (Table 2). Several variables were common across more than one workshop. For instance, all three diagrams included the variables “income” and “access to quality foods.” The research team identified the following six themes based on the variables in the CLDs: (1) built environment, (2) economic resources, (3) individual attitudes and behaviors, (4) policies and lobbying, (5) social equity, and (6) system outputs.

**Table 2:** Variables included in causal loop diagrams across three group model building workshops to explicate systems that produce food access and diet disparities

Variable	W1	W2	W3	Total loops^1^
Built environment				39
Food retail markets	–	×	×	12
Supportive built environment	–	×	–	7
Intentional greenspace	–	×	–	0
Transportation	–	×	–	0
Urban farms and community gardens	–	×	–	2
Crime	×	×	–	1
Access to quality, healthy foods	×	×	×	12
Access to culturally meaningful food	×	–	–	1
Neighborhood quality	×	–	–	0
Safety	–	×	×	4
Economic resources				21
Investment (into communities)	×	×	–	5
Community empowerment	–	×	–	3
Community knowledge and information sharing	–	–	×	0
Food prices	–	×	×	3
Disposable income for food	–	–	×	2
Resources and wealth	–	×	–	1
Income	×	×	×	5
Property ownership	×	–	–	2
Individual attitudes and behaviors				1
Diet “healthfulness”	–	–	×	0
Consumption of food away from home	–	×	–	0
Consumption of fast food	×	–	–	0
Nourishment	–	–	×	0
Disparities in time for diet-related activities	×	–	–	1
Politics, policies, and social systems				31
Community land use and control	–	×	–	2
Capitalism	–	×	–	0
Market forces (corporate lobbying)	–	×	–	1
Interests of corporations	–	–	×	1
Gentrification	–	×	×	9
Immigration policy	–	–	×	2
Local food policy	–	×	–	3
Cost of living	×	–	–	1
Food availability (upstream supply)	–	–	×	2
Disparities in perceptions of (community) buying power	×	–	–	4
Political power	–	–	×	2
Voting	–	–	×	1
Developers	×	–	–	1
SNAP and public benefits (eligibility and program access)	–	–	×	0
Emergency food relief	–	×	–	2
Social equity				15
Incarceration	–	×	–	2
Respect for differences in ancestral and cultural food contexts	–	–	×	1
Indigenous removal	–	×	–	1
Asian (immigrant) communities	–	–	×	2
Language barriers	–	–	×	0
Segregation	–	×	–	0
Redlining	×	×	–	2
Structural racism	×	×	–	1
Racism	×	–	–	2
Racial oppression	–	–	×	1
White supremacy	–	×	×	3
System outputs				26
Diet disparities	–	×	–	8
Food security	–	×	×	4
Chronic disease disparities	×	–	–	3
Health	–	×	–	1
Health/quality of life	–	–	×	2
Housing security	–	–	×	2
Poverty disparities	×	–	–	6
Climate change	–	×	–	1

^1^The column “Total loops” is the sum of the count of loops in which every unique variable within a theme appears; it is not a count of the unique loops in which any variable in the theme appears, because multiple variables within a theme may appear in the same feedback loop (e.g., food retail markets and supportive built environment are both in the same feedback loop from Workshop 2)

“–” symbol indicates that the variable was not included in a workshop's CLD; “×” symbol indicates that the variable was included in the workshop's CLD

There was a total of 43 feedback loops across the three aggregate CLDs (Supplemental Figs. 1–3). Collectively, variables in the built environment theme appeared in 39 feedback loops; the most common variables were “food retail markets” and “access to quality, healthy foods,” each of which appeared in 12 feedback loops. The variable “supportive built environment” was in 7 feedback loops. Variables from other themes that were present in many feedback loops include “gentrification” (9 feedback loops), “diet disparities” (8), “poverty disparities” (6), “investment into communities” (5), and “income” (5).

**Fig. 1:**
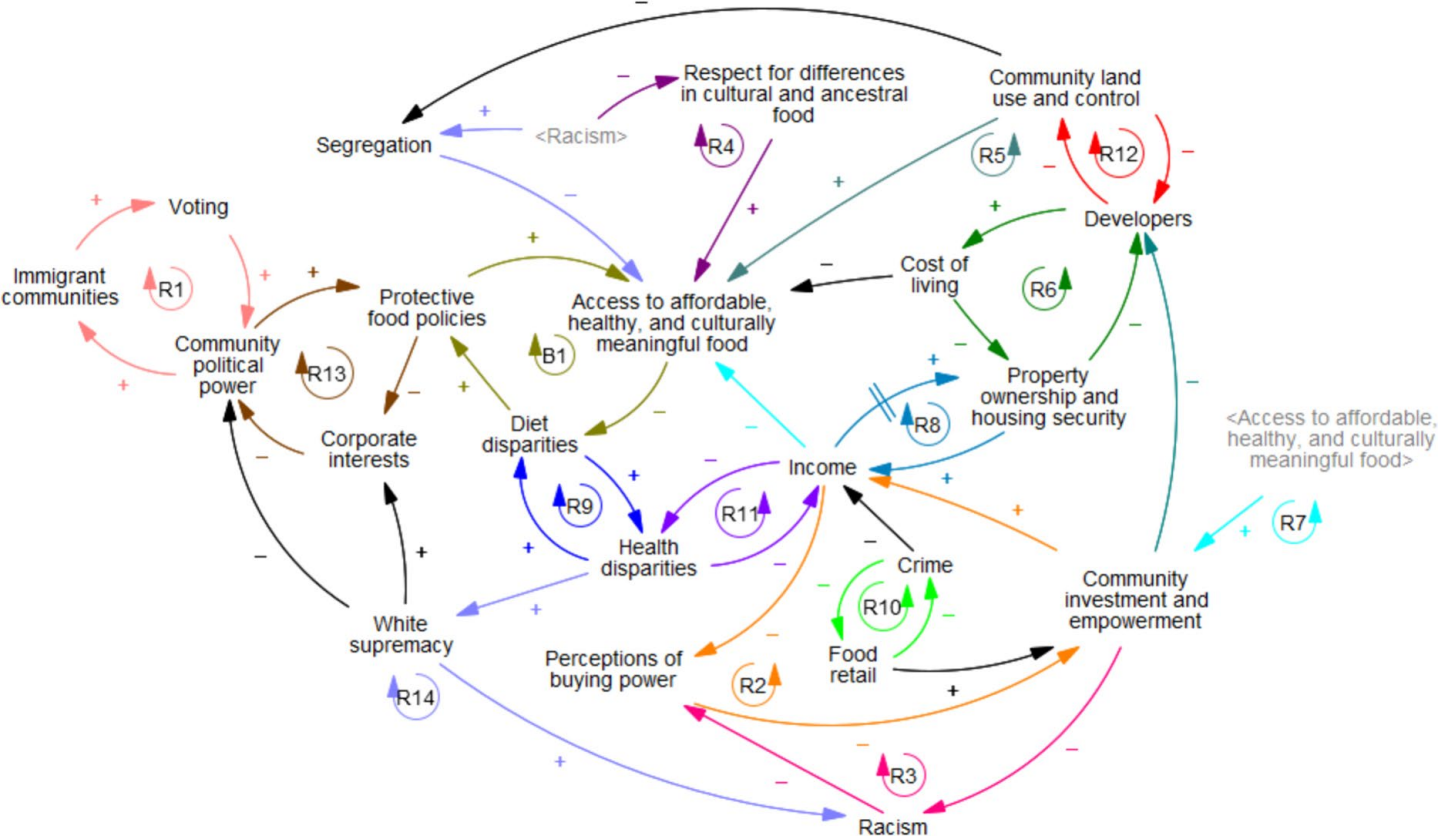
Synthesis causal loop diagram explaining food access and diet disparities in Philadelphia

The synthesis CLD (Fig. 1) includes one balancing and 14 reinforcing feedback loops. In Table 3, we present a description of each feedback loop, developed using the words of participants through session recordings and notes. The variables with the highest degree centrality—and thus the most highly connected within the system—were “access to affordable, healthy, and culturally meaningful food,” “income,” and “community investment and empowerment.”

**Table 3:** Descriptions of feedback loops included in the synthesis causal loop diagram explaining food access and diet disparities

R1	**Voting → community political power → immigrant communities**
	*Growing immigrant communities and the power of voting.* Increased voting rates allow for communities to self-govern, perhaps passing more immigrant-friendly policies. Immigrant communities are more likely to reside in places with community political power, as they may be more likely to pass immigrant-friendly policies. Larger immigrant communities allow for greater political representation and increase voting rates among immigrants
R2	**Income → disparities in perceptions of buying power → community investment and empowerment**
	*Poverty and investment disparities.* Increased disparities in poverty levels across neighborhoods lead to widening disparities in the perceptions of public and private decision-makers regarding the buying power within those neighborhoods. Differences in communities’ perceived buying power affect levels of potential community investment, as businesses may not want to open in areas they do not anticipate will be successful. Greater investment back into the community results in increased opportunities for income due to economic revitalization and a greater number of businesses in the area
R3	**Disparities in perceptions of buying power → community investment and empowerment → racism**
	*Racism and development.* Differences in communities' perceived buying power affect levels of potential community investment, as businesses may not want to open in areas they do not anticipate will be successful. Lower investment in communities leads to increased levels of racism. Increased levels of racism feed back into perceptions of neighborhoods’ buying power, as minoritized communities may be seen as having less buying power
R4	**Access to affordable, healthy, and culturally meaningful food → diet disparities → White supremacy → racism → respect for differences in ancestral and cultural foods**
	*Respect for different ancestral and cultural foods fights racial oppression and reduces diet disparities*. Access to quality, healthy foods decreases diet disparities because there are greater choices in food and greater affordability. Greater disparities in diet can lead to increases in White supremacy, as diet healthfulness is viewed through a White ethnocentric lens. Higher rates of White supremacy create a system in which there are more opportunities for racism. Decreased racial oppression allows for more celebration of different cultures and greater connections to one’s culture/ancestors and ultimately results in a greater respect for differences in ancestral and cultural food contexts. Increased respect for differences in ancestral and cultural food contexts results in greater food access for different communities because there will be more choices for food options of different cultural and ancestral backgrounds
R5	**Community investment & empowerment → developers → Community land use → access to affordable, healthy, and culturally meaningful food**
	*Community investment as a way to increase food options.* Community empowerment decreases gentrification because developers may not exploit economic opportunities for development and displacement in empowered communities. Fewer opportunities for developers to gentrify areas allow communities to maintain control over their own land and afford to stay. Increased control of the community over their own land results in greater use of the land for community-focused needs for food, improving access. More access to food allows communities not only to have more food choices, but also opportunities for business ownership, which improves community empowerment
R6	**Developers → cost of living → property ownership and housing security**
	*Gentrification and property ownership.* The increased presence of developers and redevelopment of neighborhoods results in increased property values and cost of living, including the cost of property taxes, food, or other necessities. The higher cost of living displaces existing property owners, increasing housing insecurity and making it difficult for neighborhood residents to buy homes. As community property ownership and housing security decrease, development increases further
R7	**Access to affordable, healthy, and culturally meaningful food → community investment and empowerment → income**
	*Income improves access to healthy foods.* More access to food allows communities not only to have more food choices, but also opportunities for business ownership, which improves community empowerment. Greater investment back into the community results in increased opportunities for income due to economic revitalization and a greater number of businesses in the area. Greater income improves an individual’s ability to access quality, healthy food because they can overcome access barriers like unaffordability and physical inaccessibility (e.g., having transportation if living far away from a food market)
R8	**Income → property ownership and housing security**
	*Property ownership as a source of income.* After a delay, an increase or accumulation of income allows individuals/families to buy properties and be more housing secure. Property ownership creates more opportunities for higher income through wealth accumulation (e.g., rental income, home equity)
R9	**Diet disparities → health disparities**
	*Reinforcement of diet disparities and health.* Poor health could increase diet disparities via health problems limiting physical access to food (both in purchasing and preparation). Disparities in diet quality may lead to higher rates of chronic disease and health disparities in certain populations
R10	**Crime → food retail**
	*The criminalization of hunger.* Increased crime limits the number of food retailers willing to settle in the area, to avoid theft and crime. A reduced presence of food retailers in a neighborhood causes residents to seek alternative methods (crime) to feed their families
R11	**Income → health disparities**
	*Disposable income improves health.* A greater income decreases disparities in health and overall well-being/quality of life, as you can spend more on healthy food, healthcare costs, etc. Health disparities increase poverty disparities, as those who are burdened by disease have higher healthcare costs
R12	**Community land use → developers**
	*Gentrification and community displacement.* Community empowerment decreases gentrification because developers may not exploit economic opportunities for development and displacement in empowered communities. Fewer opportunities for developers to gentrify areas allow communities to maintain control over their own land and afford to stay
R13	**Community political power → protective food policy → corporate interests**
	*Corporate interests and food policy.* Increased control of the community over their own land results in greater use of the land for community-focused needs such as urban farms or community gardens. Urban agriculture opportunities can empower communities by creating jobs, allowing a community to be self-sustaining, and growing their own foods. Local policymakers are encouraged to react to the issues that pertain most to their communities, like policies that make food more accessible
R14	**Diet disparities → Health disparities → White supremacy → racism → segregation → access to affordable, healthy, and culturally meaningful food**
	*Cycles of segregation and exclusion.* Disparities in diet quality lead to higher rates of chronic disease and health disparities. Increased health disparities cause healthfulness to be viewed through a White ethnocentric lens, increasing levels of White supremacy. Higher rates of White supremacy create a system in which there are more opportunities for racism and, ultimately, segregation. Racism and othering can reduce passage of protective food policy (e.g., access to SNAP benefits, school lunches, sugar tax), which decreases access to food and increases diet disparities
B1	**Protective food policy → access to affordable, healthy, and culturally meaningful food → diet disparities**
	*Policymaking to increase food access.* Food policy, such as restrictions on advertising/marketing, can decrease the ability of food corporations to target poor and minoritized communities to provide and promote unhealthy foods. Access to quality, healthy foods decreases diet disparities because there are greater choices in food and greater affordability. Corporate interests can destabilize community political power, as those corporations will continue to lobby and contribute to politicians who ensure ongoing political representation and influence over local policymaking

An illustrative example of the RIQ analysis that we used to verify CLD elements is in Appendix 2; the example describes inputs and quotations in support of loop R6 in the synthesis of CLD. We synthesized the loop from three reinforcing feedback loops described in two of the workshops. Illustrative quotations from two of the workshops are below:

From the developers, we see the change in the neighborhood, because as you're losing your property, developers come in and grab it up. Then there is a new building, and your neighborhood starts changing.—Participant in Workshop 1

Like [Philadelphia neighborhood] became [Philadelphia neighborhood] because [developer] decided, along with [restaurateur] to collaborate together and build up that place. You could still get a house for like $100,000 and now you can't.—Participant in Workshop 3

Broadly, the synthesized loop encapsulates a fundamentally similar process, via which real estate development accelerates neighborhood gentrification by increasing neighborhood prices. This pricing out, in turn, reduces property ownership and housing security among longer-term residents.

### Promising Policies and Interventions

Across the three workshops, participants proposed 53 unique action ideas to address diet disparities in Philadelphia (see Supplementary Table 2). The research team identified the following seven thematic clusters of action ideas: (1) built environment, (2) individual attitudes and behaviors, (3) targeted redistribution of resources, (4) safety net improvements, (5) community empowerment, (6) market interventions, and (7) others. Participants most commonly identified action ideas in the “individual attitudes and behaviors” theme (*n* = 12 action ideas), the “safety net improvements” theme (*n* = 10), and the “community empowerment” (*n* = 10).

Participants proposed action ideas targeting both upstream factors related to social determinants of health and downstream factors that were specific to food or food access. Action ideas addressing social determinants included universal health coverage, funding to increase general and low-income housing, reparations, regulations to limit gentrification, increased access to high-paying jobs, and guaranteed basic income. Generally, participants rated these action ideas as highly impactful but relatively difficult to implement. Action ideas targeting food and food systems included funding to build more supermarkets with quality foods, resources for community gardens or urban agriculture, taxes on retailers, nutrition education, and incentives for consumers to purchase nutritious foods or shop locally. Participants rated these ideas as easier to implement than those addressing social determinants, but less impactful.

## Discussion

Findings from this study yield several insights with respect to participants’ perspectives on systems that produce and maintain disparities in food access and diet in Philadelphia: First is that participants placed relatively lower emphasis on downstream variables and greater emphasis on upstream variables. The most highly connected downstream variable was diet disparities, the main outcome that participants were asked to explore. Other highly connected downstream variables were those related to both physical and economic access to affordable, healthy, and culturally appropriate foods. Key upstream variables included those related to the social, policy, and political systems in which minoritized urban communities are embedded. Among the most highly connected variables were those related to racism (e.g., White supremacy, racial oppression, racism, structural racism, redlining, and residential segregation) and community power (e.g., community land control, community investment, and empowerment).

Importantly, a variable with a high degree centrality is not necessarily a leverage point or a place in a system where a relatively small change can have an outsized impact on system behavior [30]. Identification of leverage points requires examination of the strength and directionality of causal relationships, as well as the overall structure of the system. However, several of the most highly connected variables in the synthesis CLD—and particularly the upstream variables related to racism and community power—were included in feedback structures identified during the workshops and prominent in discussions of system structure. For example, variables related to residential segregation (i.e., segregation, redlining, gentrification) were discussed in all three workshops and were involved in a high number of feedback loops. These variables warrant further qualitative and quantitative exploration to understand their role in generating, maintaining, and potentially addressing disparities.

The emphasis on upstream factors—in CLDs and descriptions of system function—adds to the large literature suggesting that the most effective means of addressing disparities in a range of health-related behaviors and outcomes (including diet) is to target fundamental social causes of disease [31], including poverty, housing security, neighborhood stability, and community power [11, 31]. Many variables identified by participants in this study sit at the nexus between the community and societal levels of influence and the built and sociocultural environment domains.

A second insight is that participants were able to identify a high number of reinforcing loops that underpin and perpetuate disadvantage among minoritized populations and communities, as well as balancing feedbacks that may prevent changes to the system. Across the three workshops, participants identified 43 unique feedback loops related to the production and persistence of disparities. These loops align with research suggesting that health disparities, and, by extension, diet disparities, are the product of a complex system [10]. Systems thinking and participatory systems methods like group model building can complement existing public health frameworks by elucidating dynamics that are sometimes overlooked but that are critical for understanding the production, persistence, and durability of health disparities.

A third insight is that both contemporary and historical examples of several of the causal connections and feedback loops identified by participants can be identified in Philadelphia. Several feedback loops described mechanisms via which specific forms of community power and inequities in power are interconnected with access to affordable, healthy, and culturally meaningful food. For example, this includes loops related to community land use and control (i.e., R5 in the synthesis model), racism (R4), and community investment and empowerment (R5, R7). Recent work by Gripper et al. (2022) illustrates a similar dynamic, in which many Philadelphia neighborhoods experienced economic devastation in the 1950s and 1990s because of population decline, White families fleeing to suburbs, and businesses leaving the area [32]. Black and immigrant communities reclaimed vacant spaces to grow their own food and, as a result, community-based organizations emerged that were focused on urban agriculture, ultimately increasing access to fresh produce [32].

Contemporary examples can also be identified for causal connections in feedback loop R5 linking real estate development, community land use, food access, and community investment and empowerment. Research that informed the city’s recent urban agriculture plan revealed that over 140 community gardens in Philadelphia, most of which were developed organically by local urban gardeners on abandoned or blighted property, were lost due to demolition by land owners, redevelopment, or other factors [33]. Similarly, an estimated one-third of the remaining active community gardens are threatened by gentrification, particularly those on tax-delinquent land [33]. Communities’ repurposing of vacant and blighted properties for community gardens exemplifies a link in R5, positing that community land use and control can improve access to affordable, healthy, and culturally meaningful foods. A contemporary example of the next relationship in R5—that improving food access can increase community investment and empowerment—is the role that local restaurants and food markets have played in opposing the development of a proposed professional basketball arena in Philadelphia’s Chinatown neighborhood [34, 35]. Community coalitions used a survey of 100 Chinatown businesses—90 of which oppose the new construction—in lobbying and political organizing efforts to oppose the proposal [35]. This political organizing also exemplifies the final posited relationship in R5: that empowered communities can act as a check on real estate development.

Though the current study is grounded in the Philadelphia context, many of the study’s insights are relevant to other urban contexts. For example, there are several overlaps between our findings and those of Freedman et al. (2022), who explored nutrition equity in Cleveland. Both studies identified variables and feedback structures related to the role that community land use plays in ensuring access to affordable and appropriate food, as well as social and political dynamics that impact communities’ fair access to land. Similarly, both studies emphasized the importance of community investment and empowerment to advance racial equity, including by creating well-paying jobs in communities and ensuring access to healthy food retail. Both studies also included feedback loops via which increases in communities’ political power and policy engagement can lead to food policies that advance equity. These similarities may represent common underlying structures that can be leveraged in efforts to build and advance food system equity across varying urban contexts.

### Strengths and Limitations

This study has several important strengths and some limitations. First, we used an established method—group model building—to engage a broad range of policy, research, and community stakeholders to define the systems that drive disparities in food access and diet between Philadelphia neighborhoods, as well as to identify systems-informed action ideas to address disparities. The synthesis CLD includes variables that are generally consistent with those found in other disparities frameworks; the main innovation, however, is that the CLD emphasizes the interrelationships between these variables and the feedback loops that perpetuate disparities. Another strength of the study is our approach to developing the synthesis CLD, particularly via the combination of thematic cluster analysis, degree centrality computation, and RIQ methods [28, 29]. This triangulation approach helped us to identify and confirm elements (i.e., variables, relationships, feedback loops) across the multiple CLDs produced in the three workshops and to ensure consistency with participants’ perspectives.

A potential limitation of the study is that we did not attempt to recruit a probabilistic sample of stakeholders in the Philadelphia food system, and as with any community-engaged research, findings reflect the unique perspectives and positionality of participants. Furthermore, we did not attempt to “empirically verify” causal links posited by participants (e.g., by identifying studies to support and quantify the causal influence of X on Y). This general limitation notwithstanding, many of the upstream variables that were most central to the synthesis CLD (e.g., those related to racism, community empowerment, income) are clearly highly relevant to Philadelphia and other cities.

Another potential limitation is that the synthesis CLD was developed by the research team and not directly verified by participants post-synthesis (though it was shared with them). This may have resulted in deviations from participants’ perspectives. However, these risks were mitigated through our use of content analysis of notes and transcripts, degree centrality, and RIQ methods in the development of the CLD. An alternative approach would be to design a follow-up workshop with return participants to directly develop the synthesis CLD or to review, consider, and refine the synthesis CLD. Such an approach, however, would increase participant burden and could also omit the perspectives of participants unable to return for a synthesis activity.

### Conclusions

Stakeholder-engaged systems thinking methods like group model building are useful for creating a shared understanding of the systems that produce and reinforce inequities in health. Findings from this study, driven by local experts, suggest that effectively addressing disparities in food access and diet will require not only targeting upstream social determinants, but also recognizing and disrupting the relationships and feedback loops that connect upstream factors, reinforce disparities and place minoritized neighborhoods and communities at ongoing disadvantage, and limit the effectiveness of policies and interventions targeting a single factor. Promising policies include those that empower communities, provide communities with mechanisms to retain and use land and other assets for their own benefit, and disrupt the multiple, reinforcing mechanisms via which racism creates and sustains health and social disparities.

## Supplementary Information

Below is the link to the electronic supplementary material.

Supplementary file1 (PDF 430 KB)

Supplementary file2 (DOCX 122 KB)

Supplementary file3 (DOCX 738 KB)

Supplementary file4 (DOCX 23 KB)
