# Supplementary material for: Participatory Systems Thinking to Elucidate Drivers of Food Access and Diet Disparities among Minoritized Urban Populations
Source: J Urban Health. 2024 Jul 24;101(6):1235–47. doi: 10.1007/s11524-024-00895-3 (PMC11652438; doi:10.1007/s11524-024-00895-3)
Supplement: Supplementary file 1 — Supplementary file1 (PDF 430 KB) [file 11524_2024_895_MOESM1_ESM.pdf]

Complex systems thinking and diet disparities in cities

Group model building workshop
May 25, 2022

Workshop Agenda

Last revised: August 30, 2023

DREXEL UNIVERSITY
Dornsife
School of Public Health

Complex systems thinking and diet disparities in cities

Drexel University
Dornsife School of Public Health
Philadelphia, PA
May 2022

Objectives 2
Modeling Team 2
Participants: 5
Planning Logistics 5
Agendas 6
Group Model Building Scripts 14

Objectives

Workshop Objectives (Explicit):

1. Convene stakeholders from policy, research, and community settings to map the local systems that produce and reinforce diet disparities in Philadelphia
2. Apply systems thinking and causal loop diagrams to diet disparities in Philadelphia
3. Identify policy priorities for reducing diet disparities in Philadelphia
4. Provide input that will inform design of an agent-based model to examine policies to reduce diet disparities in Philadelphia

Workshop Objectives (Implicit):

- Learn about and expand mental models of stakeholders around diet disparities in cities
- Identify common structures/drivers and variations among stakeholders with different expertise and perspectives related to diet disparities
- Build a cohort of policy stakeholders that can use the eventual ABM as a policy decision support tool

Modeling Team

Core Modeling Team

The core modeling team (CMT) is responsible for the design of the workshop.

Role	Team member

Modelers	
Familiarity with Stakeholders	
Scientific Objectives	
GMB Process	

Facilitation Team

The facilitation team is responsible for the delivery of the workshop itself. Facilitation team members may be a part of the core modeling team, or could be brought in for the facilitation only.

Role	Team member
Convener/Closer ***	
Community Facilitator	
Modeler Facilitator *	
Note Taker	
Process Coach *	
Reflectors	
Wallbuilder & Runner **	
Runner	

Facilitation Team Roles

Meeting Convener/Closer: Primary responsibility for starting the session, introducing participants to the exercise, making sure that participants understand the purpose of the exercise within the context of their organization or community, and introducing the facilitators. Closer has primary responsibility for bringing the session to close and thanking participants for their time.

Community Facilitator: The community facilitator's primary responsibility is to extend their social capital to help the community accept and work with the modeler facilitator. This is a person who is familiar with the local or substantive knowledge of the problem being modeled and knows the local language and community norms in cross-cultural situations. The substantive expert/facilitator should have strong group facilitation skills, some exposure to system dynamics (e.g., through the planning process and training session or workshop), and have sufficient knowledge of the topic and/or community to anticipate and mediate conflicts that might arise within the group model building session.

Modeler Facilitator: Primary responsibility for system dynamics modeling and group model building process. This is a person who is trained in systems thinking/system dynamics model with expertise teaching and leading groups in the use of systems/thinking/system dynamics. The person should also have experience facilitating groups and leading group model building sessions. If the goal of the project is to develop a simulation model, it is

expected that the modeler/facilitator also be an expert modeler and able to anticipate and address the variety issues that can arise in data and modeling.

Modeler: The modeler's primary responsibility is to build the system dynamics causal maps, models, and simulations. This is a person with expertise in system dynamics modeling and software (Vensim, IThink/Stella, etc.), formulating and entering equations, testing and analyzing the model, and running simulations for answering policy questions.

Note Taker/Time Keeper: Primary responsibility for taking notes about what is said in the workshop and notifying the facilitation team when time is short. It is overall very important to start and end on time as much as possible.

Process Coach: The process coach's primary responsibility is to observe the group process with attention to how participants are experiencing the session. This role requires someone who is able to reflect on the group process and accurately identify what is happening for participants based on observing their behavior and language. The process coach also plays an evaluation role and helps provide accurate feedback to the core modeling team about how the sessions are going. The process coach should be noticing when group dynamics begin to interfere with the process and identify potential solutions.

Production Coordinator: The production coordinator's primary responsibility is to ensure that the information collected during the exercises, which includes diagrams, group model building scripts, agenda, pictures, notes, electronic versions of diagrams, etc., are collected, appropriately archived, and made available.

Reflector: Primary responsibility for helping the group reflect on what they have done so far and recognize the issues/insights that have been developed during the modeling. This role requires someone who can speak to the relevance of the activities and insights to a larger substantive context, or to system dynamics modeling more generally.

Runner: Primary responsibility is to be available to solve logistical problems as they emerge – including technical assistance, materials, etc. These people can also be on hand to support with wall building.

Wall Builder: The primary responsibility of the wall builder is to organize products from an exercise into thematic clusters, as well as to explain the clusters to the participants in order to elicit their feedback.

Participants:

Stakeholder type	Name	Professional title	Institution
Community			
Policy			
Policy			
Research			
Community			
Community			
Community			

Planning Logistics

Space Requirements

- 1 room with 20 person capacity
- Wall space for chart paper
- Projector screen & projector
- Food and drink (coffee, etc)

Materials Needed

- Digital projector
- Fat flip chart markers (at least one per participant)
- White printer paper
- Blue printer paper
- Yellow printer paper
- Blue painter's tape
- Flip charts
- Colorful 1-in dot stickers (the kind that are used in yard sales)
- Name tags
- Cameras
- Audio recorders
- Note takers

Agendas

Summary Agenda

**Activity**	**Duration**	**Time**
Participants Arrive / Coffee	30 min	800-830
Welcome & Introductions	20 min	830-850
General presentation	40 min	850-930
Hopes & Fears	45 min	930-1015
Graphs Over Time	40 min	1015-1055
Dots	5 min	1055-1100
Break	15 min	1100-1115
Causal Loop Diagramming	1 hr	1115-1215
Presentations	30 min	1215-1245
Lunch	1.5 hr	1245-215
Model synthesis	1 hr	215-315
Break	15 min	315-330
Action Ideas	55 min	330-425
Dots	5 min	425-430
Reflection	20 min	430-450
Close	10 min	450-500

Anticipated Outputs

**Activity**	**Output**
Welcome	--
General presentation	
Hopes & Fears	List of hopes & fears
Graphs Over Time	Graphs over time<br>Clusters/themes
Dots	Ranking of relative importance of GOTs
Break	
Causal Loop Diagramming	2-3 CLDs
Presentations	--
Lunch	
Model synthesis	Synthesis CLDs<br>Identification of common structures/variables<br>Example of CLD function
Action Ideas	List of action ideas, ranked by feasibility, potential impact
Dots	Ranking of importance of variables in synthesis CLDs
Reflection	Notes on key points
Close	

Detailed Agenda

Time	Activity	Roles	Description
7:30-8:00	Room Setup		
8:00-8:30	Participants arrive		Folks will arrive and complete pre-evaluation (if not completed in advance). Coffee available.
8:30-8:50	Welcome & Introductions	Convener:	Key points:<br>• Introduction of facilitation team<br>• Introduction of participants<br>• Summary of goals and agenda for the day
8:50-9:30	Presentation	Study:<br><br>Systems approaches:	Talking points:<br>• Systems approaches in general<br>• Examples of GMB in other contexts<br>• Integrating research and policy<br><br>Goal: communicate the dual benefit of this workshop for research and for stakeholders themselves
9:30-10:15	Hopes & Fears	Community facilitator:<br><br>Wallbuilder:	**Community Facilitator** asks participants to write hopes and fears for the workshop on pieces of blue (fear) and yellow (hope) paper.<br><br>S/he highlights that one participants should write one hope or fear per paper.<br><br>Gives 5 minutes to write as many hopes and fears as can think of.<br><br>With 1 minute left, ask participants to stack their hopes and their fears with the most important ones on top.<br><br>After 5 minutes, the facilitator asks someone to share 1 hope and 1 fear.<br><br>**Wallbuilder** will take the hopes and fears and cluster them on the wall.<br><br>After 1-2 rounds of sharing, **Wallbuilder** will summarize the hopes and fears, highlighting thematic clusters, and asking for additional observations.<br><br>Example hopes and fears:<br>• Fears:<br>○ People entering and leaving the workshop to attend to other meetings, answer emails, etc.

			◦ Losing a lot of time if people are late to return from breaks<br>• Hopes:<br>◦ Everyone's opinion is valued the same and everyone feels free and open to share
1015-1020	*5-minute break*		
10:20-10:55	Graphs Over Time	Modeler Facilitator:<br><br>Wallbuilder:	The **Modeler Facilitator** introduces the graphs over time by highlighting that it's easy to come up with explanations about why we behave the way we do, but we need to really think deeper. What's true today may not have been true before...S/he provides 2 examples. The examples will highlight **three** criteria: 1) shorter and longer time horizons, 2) tangible and intangible variables, 3) different groups.<br><br>Options:<br>• Invasive vs native species<br>• Defense vs non-defense spending<br>• James Harden contract $ vs performance<br><br>The **Modeler Facilitator** will ask participants in their respective groups to draw as many graphs over time as they can in the next 5 minutes. The prompt used to facilitate this activity will be as follows:<br><br>*PROMPT:*<br><br>*Please think of a factor that influences or causes diet disparities in Philadelphia. Focus specifically on differences by race, income, or immigrant status that are socially rooted, unfair, and inequitable.*<br><br>*On each paper, draw at least two trajectories – how the factor that influences diet disparities changes over time. On the same chart, please include the trajectory that you hope will happen and the trajectory that you fear will happen.*<br><br>*For disparities, feel free to draw separate trajectories for each group or to draw one trajectory that represents the difference between groups.*<br><br>Participants have **5 minutes** to draw the graphs, but this can be monitored and extended/shortened by a few minutes if necessary. At 4 minutes, a **1-minute warning** is given, and they are asked to

			begin stacking their graphs over time with the most important/favorite on top and least important/least favorite on the bottom.<br><br>The **Modeler Facilitator** then calls a stop and then goes around using a nominal group technique where each person identifies their top graph over time and explains the graph, which is handed to the **Wallbuilder** who clusters the behavior over time graphs (BOTGs) on the wall.<br><br>The **Wallbuilder** then review the clusters and themes, highlighting uncertainty in her choices, and asks participants if there are any changes to be made in where each graph is situated.
10:55-11:00	Dots	**Modeler Facilitator**:	The **Modeler Facilitator** asks participants to take the dots they have on their tables and vote for the most important drivers of changes in diet disparities over time. They can vote 5 times for the same graph, or one for each. Colors don't matter.
11:00-11:15	*Break*		
11:15-11:30	Introduction to CLDs	**Modeler Facilitator**:	First, the facilitator will tell a story of chickens reproducing, population growth, overcrowding, and injuries.<br><br>Then, the facilitator will present 2 graphs over time to describe these dynamics – of chicken population growth and stabilization, and of injuries.<br><br>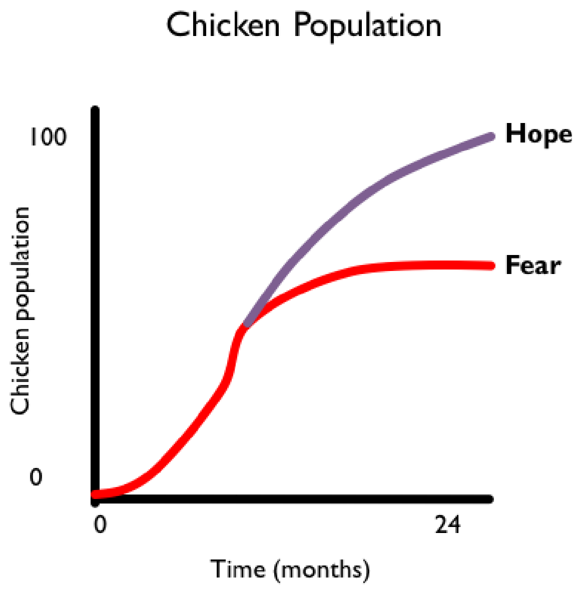

<table>
<tr>
<td></td>
<td></td>
<td></td>
<td>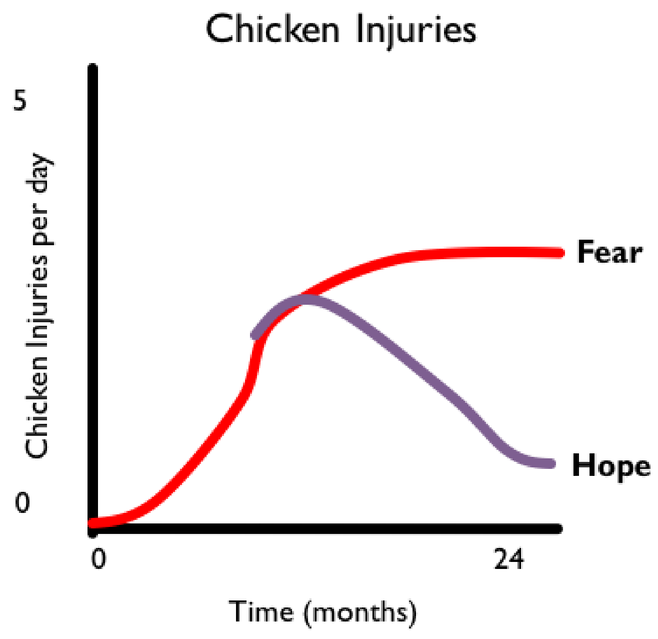

The facilitator then describes a set of feedback loops as a dynamic hypothesis of a structure that may create this behavior
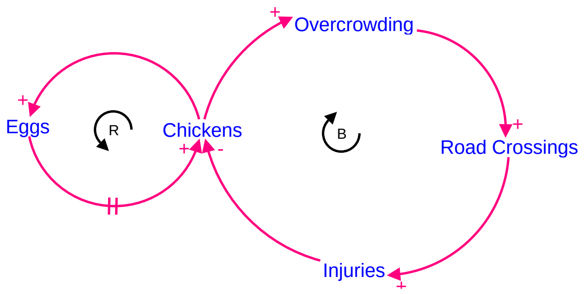

The presentation will highlight:
1. Polarity
2. Variable definition
3. Feedback loops
4. Delays

The facilitator emphasizes that the CLD is a hypothesis about the structures that could create diet disparities. S/he then asks if there are other factors or structures, and can add on other sources of injuries, other factors that reduce eggs, etc.</td>
</tr>
<tr>
<td>11:30-12:15</td>
<td>Causal Loop Diagramming</td>
<td>Modeler Facilitator:

Community Facilitator:</td>
<td>People will be assigned to subgroups of 3-4 people from the same stakeholder type (policy, community, research).

Each subgroup will work on building their own causal loop diagram that addresses the same prompt:</td>
</tr>
</table>

			**Build a causal loop diagram that explains diet disparities in Philadelphia. This can include the systems that initially produced diet disparities or that affect changes over time in diet disparities (i.e., whether disparities persist, get smaller, or get bigger).**<br><br>Prompt: What are the drivers of disparities in healthy diet in Philadelphia?<br><br>While groups work, the facilitation team will circulate, asking questions and providing guidance if folks are stuck.<br><br>With 5 minutes left CF give a 5-minute warning and ask folks to focus on consequences of target behavior, closing feedback loops.
12:15-12:45	Presentations	**Community Facilitator:**<br><br>**Modeler Facilitator:**	Teams will present their models to the larger group, focusing on highlighting feedback loops and key stories. Presentations can include narratives describing relationships in the causal loop diagram by telling a story, rooted in lived experiences, of how variables change dynamically.<br><br>*[Meanwhile **Modelers** begin synthesizing CLD with the aim of creating a draft of the synthesis causal loop diagrams]*
12:45-2:15	Lunch		*[Modelers continue synthesizing CLD]*
2:15-3:15	Model Synthesis	**Modeler Facilitator:**<br><br>**Community Facilitator**:<br><br>**Supplies**: Projector	**Modeler Facilitator** will describe how over lunch, the facilitation team reviewed the models and created a united conceptual model that links all of the CLDs that were developed in the first session.<br><br>Then, the **Modeler Facilitator** will project the CLD and describe how it was constructed using the input of the morning's CLDs, highlighting key stories and ideas.<br><br>**Modeler Facilitator** then leads a conversation with the team to review whether the model accurately captures the stories of the group, or whether there are new perspectives to be included.<br><br>**Community Facilitator** will facilitate conversations among participants and ask clarifying questions.<br><br>**Modeler Facilitator** will make revisions to the model based on proposed changes. The goal is to

			converge on a consensus view of the problem from a feedback perspective. We will revise the model until no one has adjustments or disagreements to add.
3:15-3:30	*Break*		
3:30-4:25	Action Ideas	**Community Facilitator**:<br><br>**Wallbuilder**:	The **Community Facilitator** presents on different types of system interventions, from accelerating feedback loops to creating information flows, highlighting that a change to a parameter is often the easiest but lowest leverage change we can make.<br><br>**Community Facilitator** asks participants to think about ways we can intervene in this system. She asks the small groups to come up with their top 4 system interventions, written on a white piece of paper, and plan to map these onto the model.<br><br>At the end of 5 minutes, the **Community Facilitator** asks for a volunteer group to share.<br><br>The first group should describe how they would intervene in the system, how it would impact system performance, unintended or secondary consequences. They will then propose where to put the intervention on the matrix of feasibility and potential impact.<br><br>The **Wallbuilder** places the action idea on the wall based on this recommendation.<br><br>• Introduction to action ideas (15 minutes)<br>o Now work in the same groups to identify interventions and innovations to influence the results of the systems in the CLDs.<br>o Draw each action idea on a page showing how it fits into the CLD and on a post-it.<br>• Work in groups (30 minutes)<br>• Present back ideas to larger group and place each idea in the impact/difficulty quadrants (45 minutes)<br>o X-axis – potential impact<br>o Y-axis: implementation difficulty (cost, complexity, etc.)

4:25-<br>4:30	Dots	**Community Facilitator**:	CF distributes 6 blue dots to each participant. Then asks participants to take the dots they have on their tables and vote for the **most important factors** shaping diet disparities in Philadelphia. They can vote 6 times for the same factor, or one for each.<br><br>**Focus on: Most important factors**
4:30-<br>4:50	Reflection and Closing	**Reflector**:	Reflector/s will reflect on the day's activities, focusing on the perspectives of:<br>1. Participants<br>2. Research<br>3. Modeling

Group Model Building Scripts

Scripts were used in the group model building workshop. Scripts are structured small group exercises used in group model building. These scripts are compiled in a wikibook called Scriptapedia which is intended to be a freely distributed book and easily edited to support the creation of new scripts, discussion of what works and what doesn't, and internationalization of group model building practice. Scriptapedia is available at https://en.wikibooks.org/wiki/Scriptapedia.

License

Scriptapedia is shared under the Creative Commons Attribution-ShareALike 3.0 Unported License. Anyone is free to share (copy, distribute, and transmit the work) and remix (adapt the work) under the following conditions: (1) users must attribute the work in a manner specified by the author of the content, but not so in a way that suggests that they endorse or are using adaptation, and (2) if a user alters, transforms, or builds upon another author or authors' works, users can distribute the resulting working only under the same or similar license as this one. With this understanding, any of the above conditions can be waived if the user gets permission from the copyright holder. If any or all of the elements of the work is already in public domain under applicable law, that status is in no way affected by the license. In no way are any of the following rights affected by the license: (1) users fair dealing or fair use rights, or other applicable copyright exceptions and limitations; (2) author's moral rights, and (3) rights other persons may have either in the work itself or in how the work is used, such as publicity or privacy rights. For any reuse or distribution, the user reusing or distributing the Molecule Library and its contents must make clear to others the license terms of this work. The best way to do this is by copying this page or linking to the Creative Commons Attribution-ShareALike 3.0 Unported License website.
