# Supplementary material for: Participatory Systems Thinking to Elucidate Drivers of Food Access and Diet Disparities among Minoritized Urban Populations
Source: J Urban Health. 2024 Jul 24;101(6):1235–47. doi: 10.1007/s11524-024-00895-3 (PMC11652438; doi:10.1007/s11524-024-00895-3)
Supplement: Supplementary file 2 — Supplementary file2 (DOCX 122 KB) [file 11524_2024_895_MOESM2_ESM.docx]

**Supplemental Materials 1: Illustrative example of the RIQ analysis**

*Overview of synthesis decisions informed by the RIQ analysis*

The RIQ analysis was used to develop reinforcing feedback loop R6 in the synthesis model, from the three input loops below:


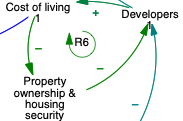


**Input Loop 1: R1, Workshop 1**


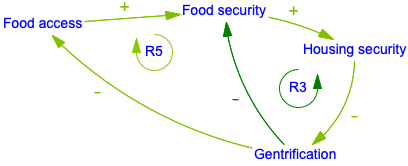


**Input Loops 2-3: R3 & R5, Workshop 1**

Quotations in the RIQ table, below, were used to help to propose the following synthesis of loops to produce R6 the synthesized CLD:

1. We combined the variables “Developers” from R1, Workshop 1 and “Gentrification” from R3 and R5. Participants defined and described the variables similarly, as the buying up of properties for new construction.
2. We combined the variable “Property Ownership” from R1, Workshop 1 and the variable “Housing Security” from R3 Workshop 3. Participants defined and described the variables similarly, as both actual displacement from homes and the threat of displacement.
3. The quotations confirm the negative arrow in the synthesis diagram from “Property ownership and housing security” to “Developers.”
4. The quotations confirm the positive arrow from “Developers” to “Cost of living.”

The synthesis loop is as follows:

**
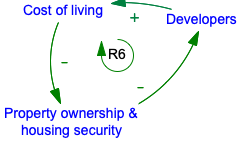
**

**Synthesis Loop R6**

**RIQ Table**

| **Feedback loop:** Developers → cost of living → property ownership and housing security | |  |
| --- | --- | --- |
| **Quotations** | **Phrases** | **CLD Elements** |
| "From the developers, we see the change in the neighborhood, because as you're losing your property, developers come in and grab it up. Then there is a new building and your neighborhood starts changing." - *Workshop 1*  "Because these properties lie vacant for so long, here comes gentrification, because that's usually what happens [when] a lot of poor people live in that community…and here comes better market systems, so the prices go up." - *Workshop 2*  "Like [Philadelphia neighborhood] became [Philadelphia neighborhood] because [developer] decided, along with [restaurateur] to collaborate together and build up that place. You could still get a house for like $100,000 and now you can't." - *Workshop 3  "*Having less [housing] security causes more displacement*. More displacement equals more vacant houses. More vacant houses equals more gentrification." -* Workshop 3 | • From the developers we see changes in the neighborhood • There is a new building • Developers come in and grab it up • [Developer] decided, along with [restaurateur] to collaborate together and build  • Vacant houses equals more gentrification | Developers |
|  | • Here comes better market systems, so the prices go up • You could still get a house for like $100,000 and now you can't | Cost of living |
|  | • Losing your property • You could still get a house • Displacement equals more vacant houses | Property ownership and housing security |
