# Supplementary material for: Participatory Systems Thinking to Elucidate Drivers of Food Access and Diet Disparities among Minoritized Urban Populations
Source: J Urban Health. 2024 Jul 24;101(6):1235–47. doi: 10.1007/s11524-024-00895-3 (PMC11652438; doi:10.1007/s11524-024-00895-3)
Supplement: Supplementary file 3 — Supplementary file3 (DOCX 738 KB) [file 11524_2024_895_MOESM3_ESM.docx]

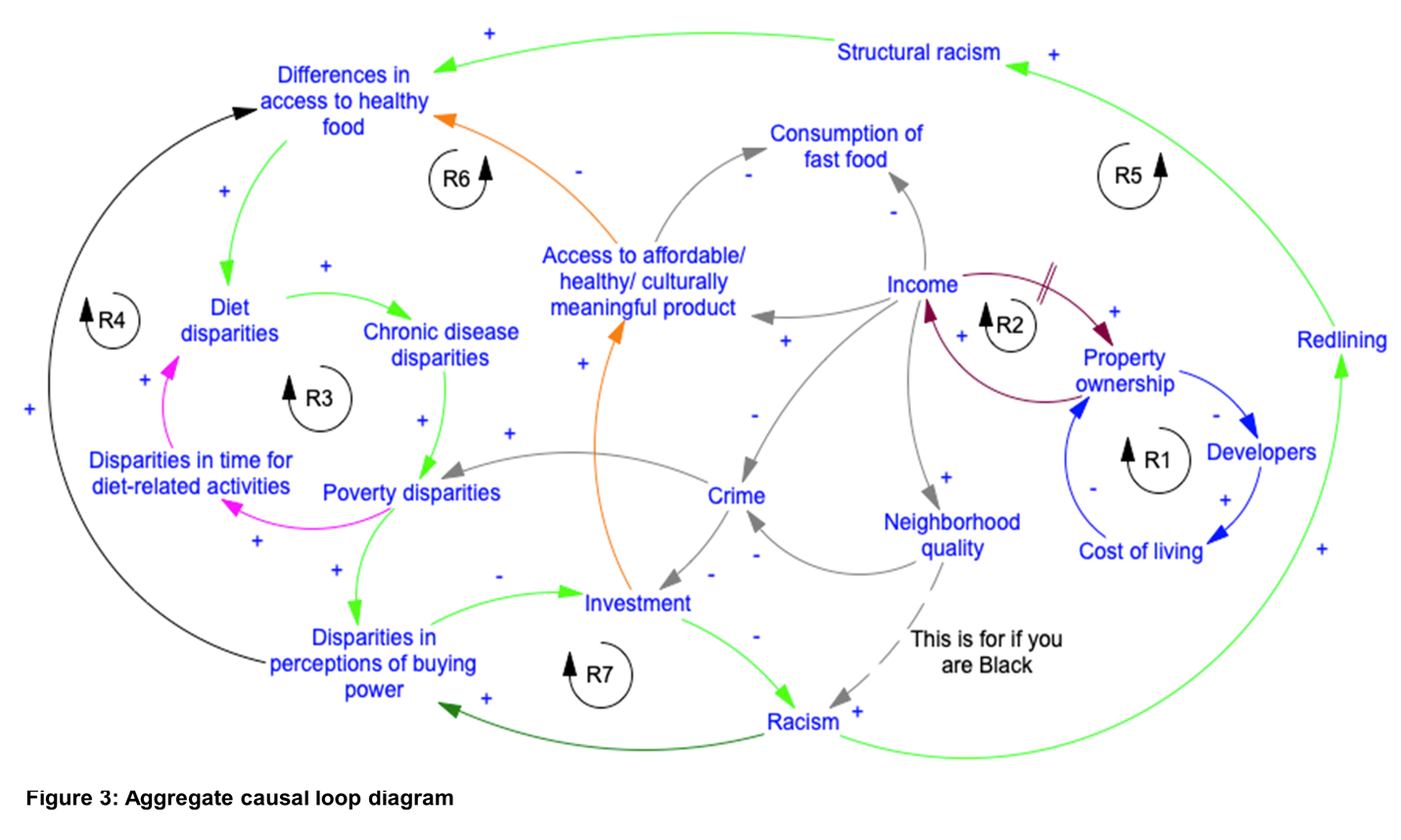


**Supplementary Figure 1: Aggregate causal loop diagram from Workshop 1**


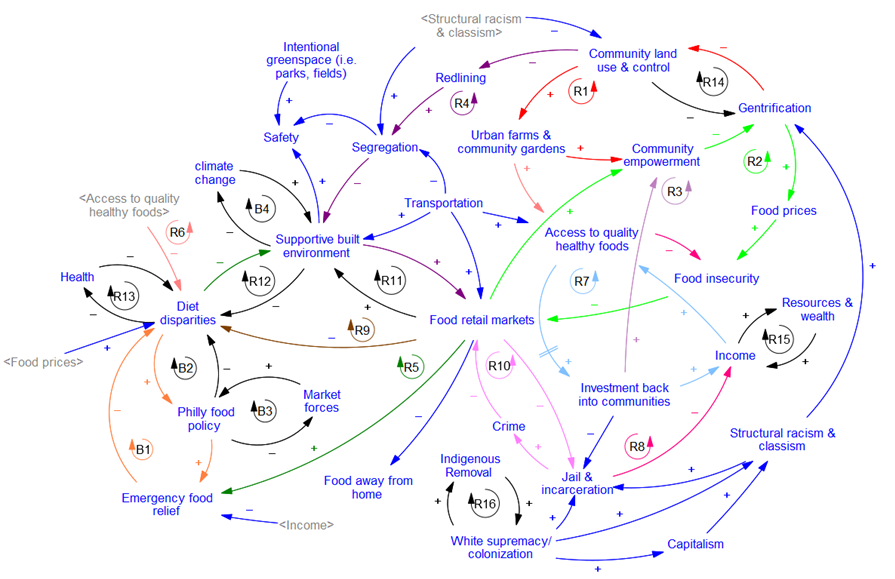


**Supplementary Figure 2: Aggregate causal loop diagram from Workshop 2**


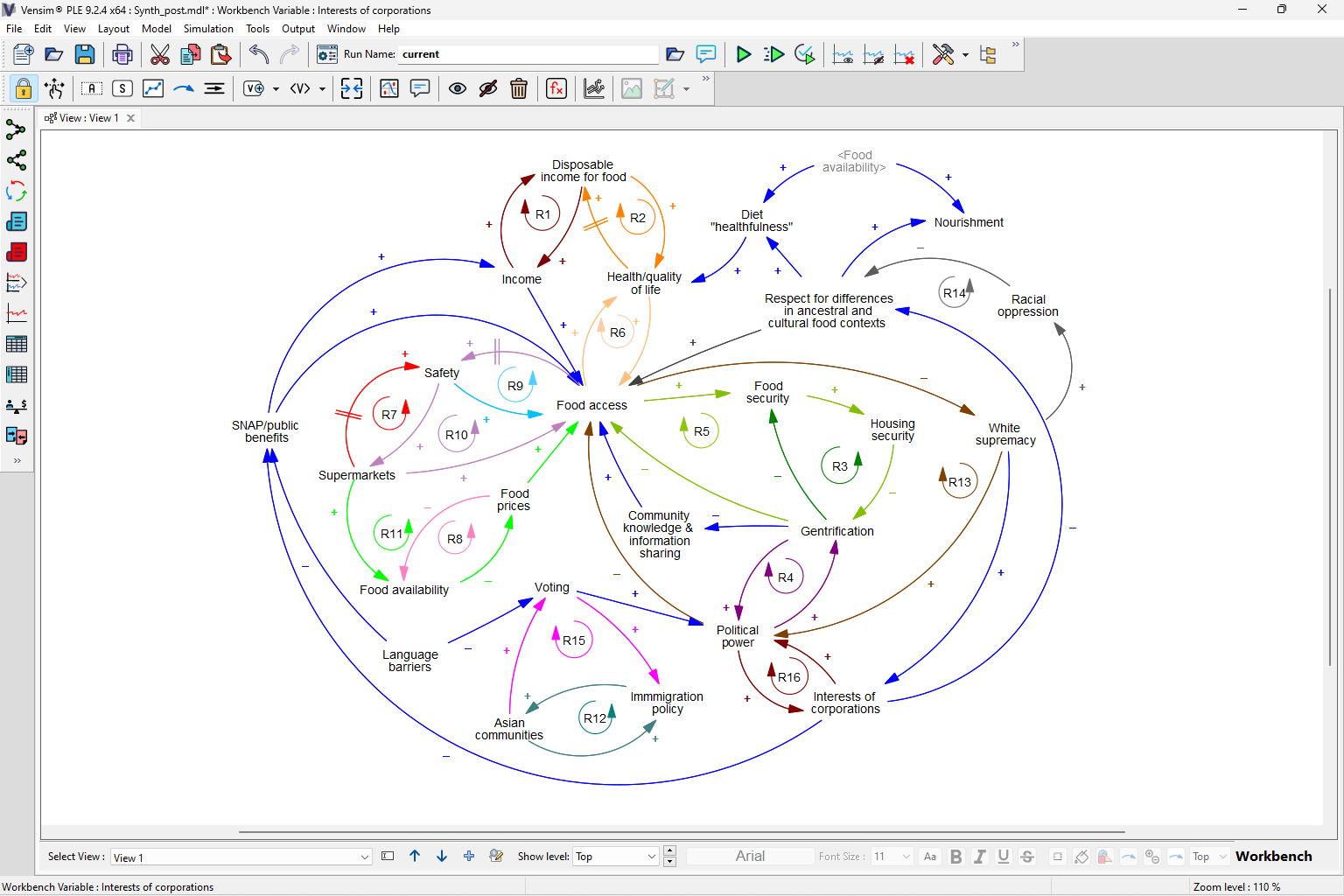


**Supplementary Figure 3: Aggregate causal loop diagram from Workshop 3**
