# Supplementary material for: Participatory Systems Thinking to Elucidate Drivers of Food Access and Diet Disparities among Minoritized Urban Populations
Source: J Urban Health. 2024 Jul 24;101(6):1235–47. doi: 10.1007/s11524-024-00895-3 (PMC11652438; doi:10.1007/s11524-024-00895-3)
Supplement: Supplementary file 4 — Supplementary file4 (DOCX 23 KB) [file 11524_2024_895_MOESM4_ESM.docx]

**Supplementary Table 1: Stakeholder Type by Workshop**

|  | **Workshop 1**  **(n)** | **Workshop 2**  **(n)** | **Workshop 3**  **(n)** |
| --- | --- | --- | --- |
| **Stakeholder Type** |  |  |  |
| Research | 1 | 3 | 1 |
| Policy | 2 | 3 | 4 |
| Community | 4 | 3 | 8 |

| **Supplementary Table 2: Action Ideas by Thematic Cluster** | | | | | | |
| --- | --- | --- | --- | --- | --- | --- |
|  | | **W1** | **W2** | **W3** | **Impact** | **Effort** |
| *Built environment* | |  |  |  |  |  |
|  | Increased urban agriculture and community gardens | - | x | - | High | Easy |
|  | Policies to mitigate effects redlining (e.g., grocery & retail financing) | x | - | - | Low | Easy |
|  | Increased government funding for infrastructure | x | - | - | High | Easy |
|  | More supermarkets with fresh, affordable food | x | - | - | High | Easy |
|  | Neighborhood gardens | x | - | - | High | Easy |
|  | Build more grocery stores | - | x | - | Low | Easy |
| *Targeted redistribution of resources* | |  |  |  |  |  |
|  | Reparations in the form of land and money | x | - | - | High | Hard |
|  | Reparations | - | x | - | High | Easy |
|  | Guaranteed basic income | x | - | - | High | Hard |
|  | Equitable income | x | - | - | High | Hard |
|  | Baby bonds | x | - | - | High | Hard |
| *Individual attitudes & behaviors* | |  |  |  |  |  |
|  | Hold free cultural/ancestral food programs in public spaces | - | - | x | High | Easy |
|  | Improving formal health and community health | - | - | x | High | Hard |
|  | Understanding folks' perceived food environment to inform programming around food literacy and skills | - | - | x | Low | Easy |
|  | Increase language access to increase agency and ability to access needs | - | - | x | High | Hard |
|  | Nutritional education | x | - | - | High | Easy |
|  | ‘Food as Medicine’ programs in local hospitals for gunshot incident survivors | - | x | - | High | Hard |
|  | Incentives to shop locally | - | x | - | High | Easy |
|  | Financial literacy education | x | - | - | High | Easy |
|  | Policies to support healthy food affordability or nutrition incentives | x | - | - | Low | Easy |
|  | Accessible nutrition and health education (food talks, health impacts of diet) | - | x | - | High | Easy |
|  | Expanding food-specific policies, such as expanding school nutrition policies | - | x | - | High | Easy |
|  | Accessible nutrition/cooking education | - | x | - | Low | Easy |
| *Safety net improvements* | |  |  |  |  |  |
|  | Universal healthcare | - | x | - | High | Hard |
|  | Livable wages | - | x | - | High | Hard |
|  | Require policy makers to do the "SNAP challenge" | - | - | x | Low | Easy |
|  | Food pantry coalitions to distribute food with choice | - | - | x | High | Hard |
|  | Affordable housing & home ownership | x | - | - | High | Hard |
|  | Improved healthcare | x | - | - | High | Easy |
|  | Reallocating government spending towards ending poverty | - | x | - | High | Hard |
|  | Universal utility security | - | x | - | High | Hard |
|  | An additional income supplement for food | - | x | - | High | Hard |
|  | Quality food at food pantries | - | x | - | Low | Easy |
| *Community empowerment* | |  |  |  |  |  |
|  | Reclaiming cultural norms (relearning, education) | - | - | x | High | Easy |
|  | Land/housing policies that protect long-term residents and abolish councilman prerogative “intervention” between gentrification and political power | - | - | x | High | Hard |
|  | Policy to support and preserve land for community use | - | - | x | High | Hard |
|  | Community land control policies | x | - | - | High | Hard |
|  | Funding to create community-led safety plans and pathways | - | - | x | Low | Hard |
|  | Criminal interventions | x | - | - | High | Hard |
|  | Community programs | x | - | - | High | Easy |
|  | Policies requiring mixed income housing availability | x | - | - | High | Easy |
|  | Policies that include community voices in neighborhood development | x | - | - | Low | Hard |
|  | Incentives for urban community gardens to act as alternative job training to reduce recidivism | - | x | - | High | Easy |
| *Market intervention* | |  |  |  |  |  |
|  | Connect food access and insecurity to economic and healthcare expenditures | - | - | x | High | Hard |
|  | Target racial oppression by changing financial structures – racial capitalism | - | - | x | High | Hard |
|  | Control high inflation that impacts food prices and food availability | - | - | x | High | Hard |
|  | Corporate accountability and regulations | - | x | - | High | Easy |
|  | Junk food tax on retailers | x | - | - | Low | Easy |
|  | Fewer farm subsidies for industrial farming | x | - | - | Low | Easy |
|  | Taxing universities and other entities who drive gentrification | - | x | - | High | Hard |
| *Other* | |  |  |  |  |  |
|  | Raise awareness of food waste sent to Philly school district meal programs | - | - | x | High | Easy |
|  | Increasing high quality jobs and skills training | - | - | x | High | Hard |
|  | Better jobs and housing | - | x | - | High | Easy |
